# Advancing Cancer Cachexia Drug Development: Leveraging Biomarkers and Functional Endpoints to Optimize Trial Design

**DOI:** 10.1002/cnr2.70613

**Published:** 2026-07-01

**Authors:** Nada O. Othman, Hadir Habib, Doaa M. Almeldin, Kyrillus S. Shohdy

**Affiliations:** ^1^ Department of Clinical Oncology Cairo University Cairo Egypt; ^2^ Department of Oncology University College London London UK; ^3^ School of Cancer Sciences University of Glasgow Glasgow UK; ^4^ CRUK Scotland Institute Glasgow UK

**Keywords:** cancer cachexia, drug development, trial design

## Abstract

**Background:**

Cancer‐associated cachexia is a complex, multistage metabolic disorder that markedly contributes to morbidity and mortality in patients with advanced cancer, yet effective pharmacological interventions are lacking. Cachexia drug development has expanded significantly in recent years, with multiple repurposed agents and novel therapies targeting specific pathways entering clinical trials. However, clinical benefit has been limited not only by a lack of promising agents but also, to a large extent, by suboptimal trial design.

**Recent Findings:**

This article reviews the current evidence to highlight the core principles required to modernize cachexia clinical trials. We emphasize that both repurposed drugs and novel agents have a role, but multimodal approaches integrating pharmacologic intervention with nutritional support and exercise are essential for therapeutic success. Early intervention, guided by predictive biomarkers such as C‐reactive protein and Interleukin‐6, and relatively short study durations focused on rapid clinical benefit are critical considerations.

**Conclusion:**

We demonstrate the importance of moving beyond weight‐based endpoints alone toward functional primary endpoints. Selection of appropriate control arms is crucial and should include multimodal interventions, while trial designs must account for patient heterogeneity and concurrent anticancer therapies.

## 1 Introduction

Cancer‐associated cachexia (CAC) is a multistage metabolic disease. The key hallmark of CAC is unintentional weight loss. Other manifestations include skeletal muscle wasting (sarcopenia), loss of fat mass, decreased food intake (anorexia), and systemic inflammation evidenced by elevated inflammatory markers such as serum C‐reactive protein (CRP). Cachexia is distinct from other malnutrition or starvation disorders as it cannot be reversed by nutritional or dietary interventions alone. CAC is a major contributor to poor performance status and unfitness for therapy and is estimated to be linked to one‐third of cancer‐related mortality [1]. The prevalence of CAC can reach up to 30% of patients with cancer [2]. However, up to 80% of patients may experience an earlier stage of cachexia or pre‐cachexia [3].

An expert consensus definition introduced in 2011 mandated unintentional weight loss exceeding 5% of baseline body weight over the preceding 6 months, or exceeding 2% in individuals with a BMI below 20 kg/m^2^, however, a wide spectrum of symptoms usually ensues before these criteria are met, a state often referred to as pre‐cachexia [4]. Consequently, the consensus adopted phase‐specific criteria comprising three progressive phases that are pre‐cachexia, cachexia, and refractory cachexia. Although weight loss serves as a clinical marker and is incorporated into the diagnostic criteria for cachexia, the syndrome is fundamentally defined by ongoing loss of skeletal muscle mass—with or without fat mass depletion—that cannot be fully reversed by nutritional supplementation alone. Accordingly, weight loss alone does not qualify for the refractory phase. Instead, this stage must be associated with general decline in performance (World Health Organization [WHO]/Eastern Cooperative Oncology Group [ECOG] performance status score of 3 or 4), and very advanced disease that is unresponsive to anti‐cancer therapy. This phase‐based classification serves an essential purpose in determining the appropriate aim and magnitude of intervention for each stage of the syndrome.

Over the past two decades, drug development in CAC followed two paths: (I) Repurposing: an approach aiming to leverage existing, approved drugs with known safety profiles (e.g., megestrol acetate, NSAIDs, and atypical anti‐psychotics); and (II) Novel targets, an approach focused on developing new agents based on specific cachexia pathways (e.g., anamorelin/ghrelin agonists, myostatin/activin inhibitors, anti‐GDF15 antibodies). The following sections discuss the clinical efficacy of both approaches.

## 2 Methods

A comprehensive search was conducted across multiple electronic databases—including PubMed/MEDLINE, Scopus, Web of Science, the Cochrane Library, and Google Scholar—from database inception through December 1, 2025. The search strategy targeted both preclinical and clinical studies evaluating interventional approaches for the management of cancer‐associated cachexia. Additionally, literature regarding general trial design and statistical considerations in palliative care was included to provide methodological context.

To supplement the narrative synthesis, a systematic search was performed in PubMed using the MeSH terms [“cancer” AND “cachexia”] filtered by “randomized controlled trial.” This query yielded 244 results, from which three studies met the specific eligibility criteria for quantitative synthesis. These studies were used to pool the odds ratio (OR) for the proportion of patients achieving a > 5% increase in body weight when treated with olanzapine compared to a control group. All statistical analyses and meta‐analytical pooling were conducted using the meta package in Stata MP 18.

## 3 Pathophysiology of CAC

Recently, cachexia has been recognized as a distinct disease entity within the carcinogenesis process rather than a mere constellation of unrelated symptoms. The hallmarks of cachexia are depletion of energy reserves with loss of muscle mass and fat, gut‐brain axis symptoms (primarily anorexia), inflammation, and neuromuscular dysfunction [4].

Extensive deep phenotyping studies [3, 5, 6], have redefined CAC as a skeletal muscle pathology driven by various metabolic mechanisms. This metabolic reprogramming is sustained by a “cachexia cascade” initiated by the primary tumor, host inflammatory responses, anorexia, and endocrinopathies. This process culminates in complex organ crosstalk involving the secretion of pro‐cachectic metabolites and cytokines [3]. A recent study [6] sought to establish a molecular basis of cachexia through transcriptomic phenotyping of skeletal muscle in patients with pancreatic and colorectal cancer. The study identified two molecular subtypes, one clinically associated with cachexia, and another that was not. The cachectic subtype was enriched with genes involved in inflammation, extracellular matrix degradation, and prothrombin activation. This molecular characterization emphasizes the role of skeletal muscle as the central target organ in cachexia.

At the molecular level, TGF‐β superfamily is one of the most well‐characterized pathways involved in cachexia pathogenesis [7]. TGF‐β members are potent secreted cytokines that activate transmembrane serine/threonine kinase receptors (type I and type II) and downstream signaling, which is mainly mediated by SMAD proteins. Myostatin and activin A are linked to skeletal muscle catabolism; both can activate activin receptor type IIB (ActRIIB) leading to the upregulation of E3 ubiquitin ligases and atrophy‐related genes (“atrogenes”) that mediate the degradation of structural muscle proteins via the ubiquitin‐proteasome system (UPS) [7].

Although GDF15 is considered a member of the TGF‐β superfamily, it exerts a unique mode of action via the glial cell line‐derived neurotrophic factor receptor alpha‐like (GFRAL), which is exclusively expressed in the central nervous system. GDF15 is produced by tumor cells as well as other host tissues, such as the liver and kidneys, primarily secreted in response to stress and chronic inflammation. Activation of GFRAL in the area postrema induces strong satiety, causing anorexia. Additionally, evidence indicates that GDF15 is implicated in other functions, including immunosuppression. This makes GDF15 an appealing target for cachexia treatment in patients with cancer [8].

Anorexia is a cardinal feature of CAC and contributes substantially to energy deficit and weight loss. Central appetite regulation is governed by hypothalamic circuits, particularly the interplay between orexigenic neurons co‐expressing neuropeptide Y (NPY) and agouti‐related peptide (AgRP), and anorexigenic pro‐opiomelanocortin (POMC) neurons [9]. Tumor‐derived and host‐derived factors, including pro‐inflammatory cytokines (IL‐1β, TNF‐α, IL‐6), GDF15, and leptin, chronically activate POMC/melanocortin 4 receptor (MC4R) signaling, suppressing appetite and promoting negative energy balance. Additionally, ghrelin—an endogenous orexigenic hormone produced by gastric enteroendocrine cells—is paradoxically reduced or functionally impaired in cancer patients, further impairing appetite and promoting catabolism. These pathways provide the mechanistic rationale for pharmacological interventions targeting ghrelin receptor agonism (e.g., anamorelin) and MC4R antagonism (e.g., TCMCB07), which are discussed in subsequent sections [2, 10].

## 4 Drug Repurposing

Drug repurposing, defined as the application of an approved agent with an established safety profile to a new therapeutic indication, represents an efficient and cost‐effective drug development strategy, particularly in conditions such as cancer cachexia where de novo drug discovery has historically been slow. In the context of CAC, repurposing has predominantly focused on agents whose known side‐effect profiles include weight gain and appetite stimulation, such as progestins, atypical antipsychotics, and antidepressants [11, 12, 13]. This section reviews the clinical evidence for the most relevant repurposed agents, with an emphasis on their efficacy in randomized controlled trials and their limitations for mainstream adoption.

For years, cachexia was perceived as a symptom rather than a distinct disease, prompting a focus on supportive care measures aimed at symptom relief. Among these, short‐term trials of progesterone analogs or corticosteroids emerged as the only pharmacological options, with only a minority of patients deriving any actual benefit. A 2013 Cochrane meta‐analysis [14] of 23 cancer trials showed that megestrol acetate (MA) was associated with a significantly higher rate of appetite improvement and weight gain (relative effect was 2.57, (95% CI: 1.48 to 4.49) and 1.55 (95% CI: 1.06–2.26), respectively) compared to placebo. However, this comes at the expense of an increased risk of thromboembolic events and edema, as well as a lack of significant improvement in quality of life. Meanwhile, a recent meta‐analysis of 13 trials showed that MA's effect is marginal and only beneficial with short courses and higher doses [15].

**FIGURE 1:**
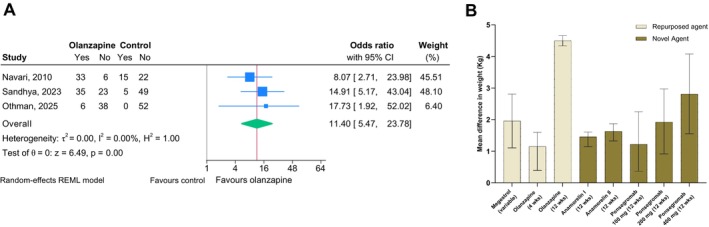
(a) Pooled odds ratio of the proportion of patients that achieved increase > 5% in weight on olanzapine compared to control in three randomized clinical trials. “Yes” indicates those who achieved > 5% weight gain and “No” for those who did not. (b) The mean difference in weight (kg) in repurposed and novel agent trials seems comparable compared to placebo controls. Error bars represent 95% confidence intervals. Number of patients; Megestrol acetate (3482), Olanzapine 4 weaks (96), Olanzapine 12 weaks (124), Ponsegromab 400 mg (95), Ponsegromab 200 mg (91), Ponsegromab 100 mg (91), Anamorelin (484 and 495).

In the past few years, several double‐blind, placebo‐controlled randomized trials have investigated the repurposing of drugs other than MA (Table 1), that are known to impact weight gain and appetite, usually as side effects [18]. Among these classes of drugs are olanzapine and mirtazapine. One double‐blind placebo‐controlled randomized trial conducted at our center investigated mirtazapine and did not show significant improvement in appetite, body weight or handgrip strength compared to placebo [13]. Regarding olanzapine, there are three randomized trials identified in our systematic review of the literature [12, 16, 17] investigating olanzapine for an extended period (> 4 weeks) for CAC. The most recent trial conducted by our group recruited 164 patients randomized to either olanzapine 5 mg daily for 4 weeks or placebo [12]. The trial was designed to test the effect of olanzapine on anorexia as the primary endpoint. In addition, functional endpoints were included, such as handgrip strength and quality of life. Another study by Sandhya et al. [16] used olanzapine at a lower dose (2.5 mg) and for extended period (12 weeks). Although there are significant differences in the eligibility criteria, a clear benefit is still observed in weight metrics. Olanzapine showed a pooled odds ratio 3.75 for achieving > 5% weight gain compared to control (Figure 1a). We also demonstrated that the mean difference in weight in both repurposed and novel agent trials is comparable when compared to placebo controls (Figure 1b).

**TABLE 1:** Characteristics of randomized controlled trials investigating repurposed drugs for cancer cachexia.

Study	Design (*n*)	Country	Intervention	Initial US FDA approval	Key endpoints
Othman et al. [12]	Double‐blind RCT (159)	Egypt	Olanzapine 5 mg (4 weeks)	1996 (atypical antipsychotic)	Appetite, BW, handgrip strength
Sandhya et al. [16]	Double‐blind RCT (124)	India	Olanzapine 2.5 mg (12 weeks)	1996 (atypical antipsychotic)	BW, appetite
Hunter et al. [13]	Double blind RCT (120)	Egypt	Mirtazapine 15 mg for 8 weeks	1996 (antidepressant)	Appetite, BW, handgrip strength
Mehrzad et al. [11]	Double‐blind RCT (70)	Iran	Pentoxifylline for 2 months	1984 (vasodilator agent)	BW, arm circumference
Navari et al. [17]	Randomized Trial (80)	USA	Olanzapine 5 mg for 8 weeks	1996 (atypical antipsychotic)	Appetite, BW

Abbreviations: BW: body weight; RCT: randomized placebo‐controlled trial.

Taken together, the available randomized evidence, including three trials and our pooled analysis demonstrating an odds ratio of 3.75 for achieving > 5% weight gain, supports olanzapine as the most evidence‐based pharmacological repurposing option currently available for CAC. Its incorporation into the 2023 ASCO guidelines [19] further validates this position. Nonetheless, olanzapine should not be considered a definitive treatment: its effects are predominantly on appetite and weight, with limited impact on muscle function or quality of life in some studies, and questions regarding long‐term metabolic tolerability remain. The clinical evolution of olanzapine use in cachexia reflects a broader lesson: repurposed agents offer a pragmatic near‐term solution, but their limited mechanistic specificity constrains the depth of benefit achievable.

## 5 Specific Cachexia Targets

One of the earliest CAC drugs was anamorelin (a selective, orally active ghrelin receptor agonist) originally developed by Novo Nordisk in 1999. Two Phase III trials were reported in 2015 (ROMANA I and II) showing improvement in weight gain but without clear improvement in functional endpoints, namely handgrip strength [20]. The drug is only approved in Japan and received a refusal of marketing authorization from the EMA on the grounds of its marginal effect on weight and lack of effect on handgrip strength or quality of life [21].

Anti‐GDF15 antibodies are another class of drugs that gained much attention within the cachexia drug development pipeline, as previously discussed. Pfizer's ponsegromab is the first‐in class antibody to progress to a phase II trial, which showed promising efficacy signals. Visugromab (CTL‐002) is another agent that followed, with a phase II/III randomized trial expected to open soon. In addition, visugromab has shown promising signals for overcoming resistance to immune checkpoint inhibitors when added to nivolumab [22]. Meanwhile, the AstraZeneca anti‐GDF15 antibody (AZD8853) did not show a pharmacodynamic effect nor sustained GDF15 suppression, prompting the termination of its early‐phase development [23]. Other anti‐GDF15 agents are still in preclinical development; for example, FL‐501 has showed a two‐fold increase in half‐life and a 50% decrease in clearance compared to ponsegromab [24].

Other pharmacotherapy agents currently in clinical development are melanocortin type 4 receptor antagonists (e.g., TCMCB07) and JAK inhibitors (e.g., ruxolitinib) [25, 26]. The phase I trial of TCMCB07 (developed by Endevica Bio) has been completed and a phase II trial is ongoing [27].

Collectively, the evolving landscape of mechanism‐specific cachexia therapies reflects both the scientific maturation of the field and its ongoing clinical challenges. Anti‐GDF15 antibodies (ponsegromab, visugromab) represent the most advanced pipeline, with promising Phase II efficacy signals and active Phase III development, though robust functional endpoints remain a gap. Anamorelin established the proof‐of‐concept for ghrelin‐axis targeting but illustrated the regulatory importance of multi‐domain efficacy. Emerging agents such as MC4R antagonists and JAK inhibitors broaden the therapeutic toolkit. A recurring theme across all these agents is that mechanistic specificity, while scientifically sound, must translate into clinically meaningful, patient‐centered functional improvement to achieve regulatory approval. The integration of these novel agents into multimodal frameworks combining nutritional and exercise interventions remains a critical unmet priority.

## 6 Challenges in Cachexia Trial Design

### 6.1 Optimal Endpoints

The key challenge in the design of CAC trials is the lack of optimal endpoints. Weight gain either an absolute or relative metric (e.g., the proportion of patients with > 5% gain) is the most common endpoint. This is usually combined with functional endpoints such as handgrip strength and improvement in appetite. A systematic review of 80 cachexia trials showed that appetite and dietary intake were primary endpoints in 15 and 7 trials, respectively [28]. A recent bibliometric analysis [29] of 191 clinical trials on cancer cachexia from 1995 to 2024 showed that the most common outcome measures in cachexia trials are molecular biomarkers and body weight.

**FIGURE 2:**
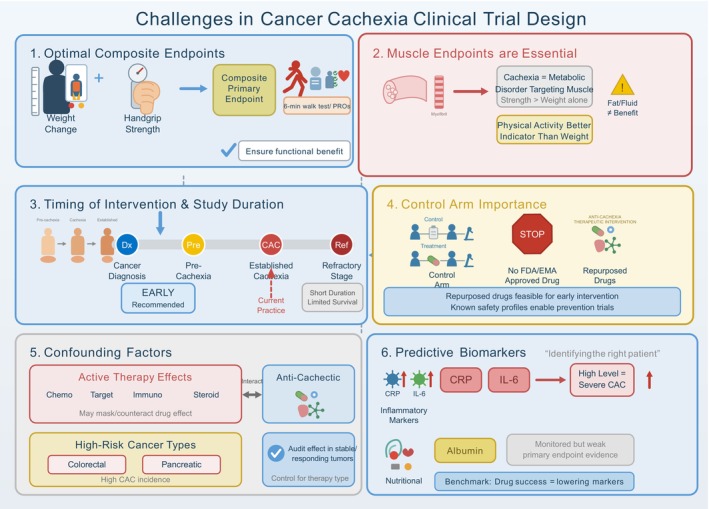
A schematic summary of the key challenges facing the cancer cachexia trial design and potential solutions. For example, the integration of objective body composition metrics (Lean Body Mass) with functional assessments to ensure that weight gain represents quality tissue accrual. A hierarchy of patient‐centered outcomes, ranging from physical performance (6‐Minute walk test) to validated patient‐reported outcomes (PROs), providing a holistic view of therapeutic efficacy.

Weight gain and/or lean body mass (LBM) gain are universally accepted endpoints for cachexia trials but must be rendered clinically relevant through the definition of a meaningful effect size. This leads to the challenging question of what constitutes a meaningful effect. While there is no universally validated threshold, several trials have used stabilization of body weight or a > 5% gain in lean body mass over 3 months as an operationally meaningful target, consistent with the magnitude of change associated with functional improvement in muscle wasting conditions [30, 31]. This threshold should be contextualized within each trial's eligibility criteria and the expected rate of muscle loss in the target population. We have already seen an instance where regulatory authorities refused to use LBM gain as the sole indicator of efficacy in the case of anamorelin necessitating positive functional endpoints [21]. We believe the primary endpoint should be a composite of weight gain and a functional measure to ensure the benefit is reflected in patient‐centered functional improvement and to confirm the intervention induces useful muscle accrual rather than just fat or fluid retention (Figure 2).

It is important to distinguish between functional endpoints and patient‐reported outcomes (PROs) such as anorexia. Appetite‐based PROs and food intake metrics, including dietary recalls and caloric intake diaries, provide complementary but not fully correlated information, as patients may maintain forced oral intake despite reporting poor appetite. Objective functional endpoints such as handgrip strength and physical performance (6‐min walk test or stair climb power [SCP]) remain more direct measures of functional capacity, and both categories should be pre‐specified separately in trial designs (Figure 2).

Lean body mass (LBM) and skeletal muscle index (SMI) are the most commonly employed body composition endpoints in cachexia trials, yet their measurement is methodologically heterogeneous across studies [32, 33, 34]. Dual‐energy X‐ray absorptiometry (DEXA) and computed tomography (CT) cross‐sectional analysis at L3 vertebral level are currently considered the most accurate methods for quantifying skeletal muscle mass, with CT offering the advantage of being available from routine staging scans [32]. Bioelectrical impedance analysis (BIA) is more feasible in clinical settings [35] but is susceptible to hydration status confounding, a significant limitation in patients receiving corticosteroids or experiencing ascites or edema. Magnetic resonance imaging (MRI) offers the highest precision but is rarely practical in clinical trials. These methodological differences limit cross‐trial comparisons and highlight the need for standardization. It is critical to distinguish between body composition metrics (which serve as diagnostic/surrogate endpoints) and functional endpoints: a functional endpoint captures a clinically meaningful patient‐centered ability, such as walking distance, stair climbing power, or grip force generation. The two categories are complementary and should ideally both be pre‐specified in trial protocols.

### 6.2 Muscle Endpoints Are a Must

We have discussed previously that emerging evidence indicates that cachexia is a metabolic disorder with skeletal muscle as the primary target tissue. It is reasonable to argue that functional endpoints that evaluate muscle strength or overall physical activity might be better indicators than a mere increase in weight [36]. However, some of the key cachexia trials seem to overlook the importance of functional endpoints. The anamorelin randomized trials included handgrip strength as a co‐primary endpoint and the drug did not show significant improvement in that metric [20]. This was one of the key reasons for the EMA refusal of its marketing authorization [21].

There are three phase II/III clinical trials investigating anti‐GDF15 antibodies, two testing ponsegromab and one testing visugromab. Notably, the primary endpoints did not include a functional endpoint in any of these three trials. The completed phase II ponsegromab trial included digitally recorded non‐sedentary physical activity as a secondary endpoint. The pivotal phase III of ponsegromab, which is due to open soon, was designed with two co‐primary endpoints: change in body weight from baseline at 12 weeks and improvement in anorexia measured by the Functional Assessment of Anorexia/Cachexia Therapy (FAACT) 5‐item anorexia symptom scale score.

Relying exclusively on weight gain as the primary endpoint may undermine the competitive positioning of novel agents in future regulatory and health technology assessments. Since several lower‐cost repurposed drugs, including olanzapine and megestrol acetate, have already demonstrated significant weight gain versus placebo in randomized trials (Figure 1b), a novel agent that achieves only comparable weight gain without demonstrating additional functional benefit would struggle to justify its preferential use over established alternatives. Functional endpoints, particularly objective measures of muscle performance, would provide the differentiation needed to support approval and adoption.

In summary, muscle‐specific functional endpoints are not merely desirable additions to cachexia trial design; they are increasingly a prerequisite for regulatory approval and for demonstrating that an intervention confers clinically meaningful benefit beyond adipose or fluid accrual.

### 6.3 Timing of Intervention

The central rationale for earlier intervention is well‐established in the cachexia literature: skeletal muscle wasting, once advanced, is difficult to reverse in the context of ongoing tumor burden and limited anabolic reserve [4, 5, 37]. Intervening at the pre‐cachexia stage, before irreversible muscle protein depletion, offers a mechanistically superior window for therapeutic effect (Figure 2). Several prospective cohort studies [5, 6] have demonstrated that significant muscle loss is already detectable at cancer diagnosis, particularly in pancreatic and colorectal cancer, supporting the case for screening‐triggered early intervention.

Detection of pre‐cachexia in clinical practice currently relies on a combination of unintentional weight loss (< 5% over six months), reduced dietary intake, and elevated inflammatory markers (e.g., CRP > 10 mg/L or elevated IL‐6) [38, 39]. Emerging approaches include CT‐derived skeletal muscle index at cancer diagnosis, which can identify patients at risk before overt weight loss occurs. Integrating routine body composition assessment into oncological staging protocols represents a feasible and clinically actionable strategy for identifying high‐risk patients eligible for preventive interventions [5].

To date, the majority of clinical studies have introduced interventions at late stage, when the cachexia is already established. Meanwhile, early intervention refers specifically to the pre‐cachexia stage identified through screening or shortly after cancer diagnosis. Repurposed drugs may be more feasible to deploy in this window owing to their established familiarity and safety data, potentially preventing or dampening the progression toward overt CAC. In contrast, novel agents could be reserved for established disease. This framework, however, presupposes a detectable pre‐cachexia window, which does not exist for all patients. Cancers frequently diagnosed at advanced stages, notably pancreatic and upper gastrointestinal malignancies, often present with measurable muscle loss already at diagnosis. Stratification should therefore be guided by cachexia stage and biomarker profile rather than by time from diagnosis alone.

It is noteworthy that screening biomarkers to identify high‐risk patients for CAC are currently lacking. These high‐risk patients might benefit from preventive strategies, as the reversal of advanced muscle wasting in a patient with limited life expectancy and a high tumor burden is a formidable endeavor.

### 6.4 Duration of the Study

Study duration is a key factor in cachexia research, as patients with cachexia often have a limited life expectancy, which necessitates shorter follow‐up periods to capture meaningful clinical benefits and symptom relief. While brief trials align with the goal of rapid symptom relief and quality‐of‐life improvement, longer studies remain essential for interventions with delayed onset, for assessing the durability of appetite‐stimulating effects, or for measuring long‐term outcomes [40]. However, extended trials in palliative care face significant hurdles; high attrition rates driven by disease progression, patient withdrawal, and treatment complications frequently compromise data integrity [41].

A practical framework for calibrating trial duration involves distinguishing between the temporal dynamics of endpoint categories. Subjective endpoints, particularly appetite‐related PROs, tend to respond rapidly and are therefore well‐suited to shorter trial windows of 4 to 8 weeks. In contrast, objective endpoints, including changes in lean body mass, handgrip strength, and 6‐min walk distance, require longer exposure periods to detect clinically meaningful differences, typically 12 weeks or more. Future trial designs should therefore pre‐specify the expected trajectory of each endpoint category and power the study accordingly.

### 6.5 The Importance of the Control Arm

What constitutes the true standard of care in cancer cachexia? No drug has received U.S.A. FDA or EMA approval for the treatment of anorexia in cancer cachexia. The 2023 ASCO guidelines [19] incorporated olanzapine as an option based on three randomized trials, that was further supported by Othman et al. study [12] (Figure 1a). Conversely, ESMO recommends that corticosteroids or progestins ‘may be’ used to improve appetite [42], whereas NCCN lists corticosteroids as optional, excludes progestins, and advises against any pharmacological treatment [43].

In the absence of an approved pharmacological standard of care, we propose that the control arm in future cachexia trials should constitute best supportive care (BSC), operationally defined as a multimodal package encompassing: (i) individualized dietary counseling provided by a registered clinical dietitian; (ii) active management of nutrition‐impact symptoms (nausea, early satiety, dysgeusia, dysphagia); (iii) structured, supervised exercise incorporating resistance and aerobic components where feasible; and (iv) psychosocial support addressing the behavioral and psychological dimensions of anorexia. This definition sets a higher bar for comparative efficacy and aligns with the evidence supporting multimodal cachexia management.

Regarding olanzapine, although short‐term trials demonstrate meaningful improvements in appetite and weight, concerns regarding its long‐term tolerability warrant attention. Adverse effects may accumulate with prolonged use, particularly in a medically vulnerable population. Giusti et al. recently raised these questions in a commentary highlighting the discrepancy between short‐term efficacy signals and unresolved long‐term safety questions in the oncological setting [44].

### 6.6 Confounding Factors; Active Anti‐Cancer Therapy and Disease‐Related Factors

Patients on active chemotherapy, targeted agents, or immunotherapy undergo ongoing modulation of inflammation and other biological parameters that can mask or counteract an anti‐cachectic drug's effect. Conversely, some of the novel therapies (e.g., anti‐GDF15) can synergize with specific classes of drugs, such as immunotherapy [22]. In either case, significant attention should be directed toward the type of treatment received during anti‐cachectic intervention as potential source of bias. Even some of the well‐established repurposed agents (e.g., steroids and NSAIDs) could interact with or complicate anti‐cancer treatments. It is worth auditing whether the intervention effect is limited to patients whose tumors are stable or responding. A successful anti‐cachexia drug should ideally work regardless of tumor progression. Furthermore, effects may differ vastly between patients with early versus advanced stages, or across different tumor types [25].

### 6.7 Predictive Biomarkers: “Identifying the Right Patient”

Predictive biomarkers are integral to the modernization of cachexia trial design for three reasons. First, they enable the identification and enrichment of patient populations most likely to respond to a given intervention, increasing statistical power and reducing sample size requirements [30]. Second, they provide pharmacodynamic evidence of target engagement, supporting the mechanistic validity of the trial [22]. Third, they can serve as eligibility criteria that reduce patient heterogeneity—one of the principal sources of null results in cachexia trials to date. Their integration into trial design therefore represents a strategic, not merely scientific, priority [39, 45].

Inflammatory markers, particularly C‐reactive protein (CRP) and Interleukin‐6 (IL‐6), are among the most frequently investigated markers in cancer cachexia [45, 46]. Because systemic inflammation often correlates with disease severity, many trials utilize these markers as benchmarks to evaluate drug efficacy, either by measuring their reduction or by demonstrating clinical benefit despite high baseline levels [46]. Conversely, while nutritional markers such as albumin are routinely monitored, current evidence does not support their use as primary clinical endpoints [30].

Regarding novel biomarkers, serum GDF15 has emerged as a promising marker, particularly given the growing interest in novel anti‐GDF targeted agents. In the Phase II trial for ponsegromab, the majority of screened patients exhibited elevated serum GDF15 levels [47]. Interestingly, baseline GDF15 levels did not appear to impact clinical efficacy, which likely informed the decision to remove this specific eligibility criterion from the ongoing pivotal Phase III trial. Nevertheless, monitoring GDF15 remains potentially valuable for identifying patients with pre‐cachexia or those at high risk who may benefit from preventive interventions [47].

## 7 Patient Heterogeneity and Multimodal Approach

Patient heterogeneity represents a major confounding challenge in cachexia trials. Patients enrolled in CAC trials differ substantially in tumor type, stage, degree of systemic inflammation, baseline nutritional status, and concurrent anticancer therapy. This heterogeneity can dilute treatment effects or create spurious subgroup findings. Future trials should consider stratified randomization by cachexia stage (pre‐cachexia vs. established cachexia), tumor type, and baseline inflammatory markers (e.g., CRP or IL‐6), and pre‐specify subgroup analyses accordingly. Adaptive trial designs, which allow for pre‐planned interim analyses and sample size re‐estimation based on accumulating data, may be particularly well‐suited to this heterogeneous population.

With respect to multimodal approaches, the evidence strongly suggests that pharmacological intervention alone is insufficient for meaningful and durable gains in muscle mass or functional status [48]. A successful anti‐cachexia regimen should integrate pharmacotherapy with resistance‐based exercise and nutritional support, ideally delivered via a structured, supervised program. Trials should therefore be designed to test multimodal combinations, rather than single agents against placebo, to more faithfully reflect the clinical standard of care and maximize the probability of detecting clinically meaningful effects.

## 8 Conclusions

Cachexia drug development has seen major expansion with the introduction of several novel agents into clinical testing. We summarized in (Figure 2) the key challenges for cachexia trial design structured around six critical areas. We argue that success revolves not just on the novel compound's biologic relevance, but on a rigorous and appropriate trial design that effectively addresses the syndrome's complexity.

Both repurposed drugs and novel agents have a role, but the clinical trial design needs modernization. A successful anti‐cachexia regimen will likely be a multimodal approach, combining a potentially effective drug intervention with nutritional support and exercise, targeting patients early based on robust biomarkers. Future trials may need to compare novel agents against the best current available supportive care using composite endpoints that are clinically relevant.

## Author Contributions

**Doaa M. Almeldin:** conceptualization, writing – original draft, visualization, methodology. **Hadir Habib:** conceptualization, writing – original draft, visualization, methodology. **Kyrillus S. Shohdy:** conceptualization, writing – review and editing, validation, supervision. **Nada O. Othman:** conceptualization, writing – original draft, writing – review and editing, formal analysis, data curation, supervision.

## Funding

K.S.S. was supported by Cancer Research UK core funding to the CRUK Scotland Institute and CRUK Clinical Academic Training Programme Award (SEBCATP‐2023/100009). Funding bodies and sources played no role in the design of the study, data collection, analysis, interpretation, nor writing the manuscript.

## Conflicts of Interest

The authors declare no conflicts of interest.

## Data Availability

Data sharing not applicable to this article as no datasets were generated or analysed during the current study.
